# Protocol to study the impact of breast cancer on colonization resistance of mouse microbiota using network node manipulation

**DOI:** 10.1016/j.xpro.2025.103618

**Published:** 2025-02-06

**Authors:** Ana Laura Cano-Argüelles, Alejandra Wu-Chuang, Lourdes Mateos-Hernandez, Lianet Abuin-Denis, Apolline Maitre, Janet Avellanet, Arlem García, Dasha Fuentes, Alejandro Cabezas-Cruz

**Affiliations:** 1 Parasitology Laboratory, Institute of Natural Resources and Agrobiology of Salamanca (IRNASA, CSIC), Cordel de Merinas, 40-52, 37008 Salamanca, Spain; 2 Anses, INRAE, Ecole Nationale Vétérinaire d’Alfort, UMR BIPAR, Laboratoire de Santé Animale, 94700 Maisons-Alfort, France; 3 Animal Biotechnology Department, Center for Genetic Engineering and Biotechnology, Avenue 31 between 158 and 190, P.O. Box 6162, Havana 10600, Cuba; 4 INRAE, UR 0045 Laboratoire de Recherches Sur Le Développement de L’Elevage (SELMET-LRDE), Corte, France; 5 EA 7310, Laboratoire de Virologie, Université de Corse, Corte, France; 6 Center of Molecular Immunology (CIM), Calle 15 esq. 216, Atabey, Playa, Havana, Cuba; 7 National Center for Laboratory Animal Breeding (CENPALAB), Calle 3ra # 40759 entre 6ta y carretera de Tirabeque, Rpto La Unión, Boyeros, Havana, Cuba

**Keywords:** Cancer, Microbiology, Model Organisms, Systems biology

## Abstract

Network analysis is a powerful tool for investigating complex interactions between different microbial taxa within a community. We present a protocol to study the gut microbial community in a mouse model of breast cancer using a network-based approach. Here, we describe the procedures for tumor cell production and inoculation and 16S rRNA data processing. We then detail steps for constructing co-occurrence networks based on correlations between microbial abundances.

For complete details on the use and execution of this protocol, please refer to Wu-Chuang et al.^1^

## Highlights

• Guidance for establishing a mouse model of breast cancer
• Protocol for downstream analysis of the 16S rRNA sequencing from mouse fecal samples
• Steps to study the bacterial community assembly using a network-based approach
• Procedure for functional trait prediction of the mouse microbiome

## Abstract (editor highlights)

Publisher’s note: Undertaking any experimental protocol requires adherence to local institutional guidelines for laboratory safety and ethics.

## Graphical abstract

**Figure:**
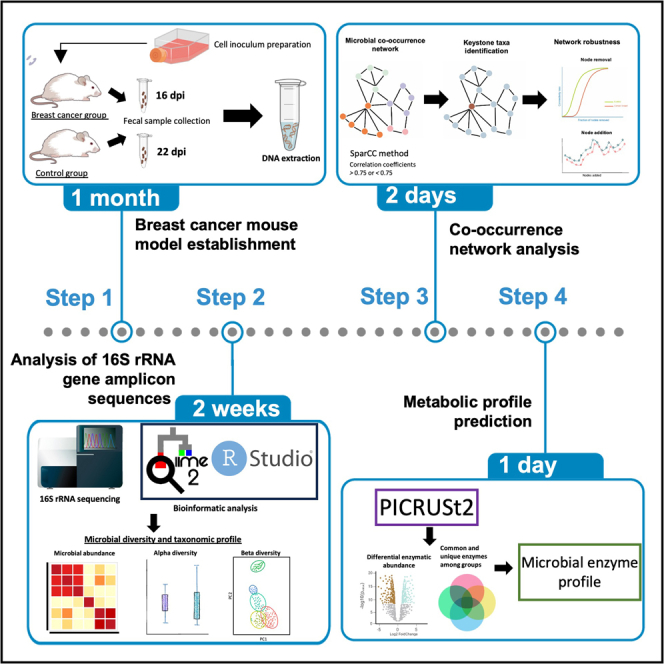

## Before you begin

This protocol outlines the specific procedures to investigate the gut microbiome of a breast cancer mouse model.

First, we describe how to produce the mouse model by inoculating tumor cells in mice and collecting the fecal samples for further microbiome analysis. Next, we present the steps for processing the 16S rRNA sequences and building the co-occurrence networks. Additionally, we conduct diversity analysis, robustness tests, and metabolic profile predictions to elucidate the impact of breast cancer on mouse microbiome dynamics.

### Institutional permissions

All preclinical studies were performed in accordance with National and International Guiding Principles for Biomedical Research Involving Animals. All procedures were reviewed and approved by the Institutional Animal Care and Use Committee (Ethics Committee) of National Centre for Laboratory Animal Breeding (CENPALAB, Cuba), with permit number 24/22. National guidelines and regulations must be followed, and relevant institutional permission must be acquired prior to commencing the following protocol.

### Cell culture and inoculum preparation

**Timing: 3 days**

These steps outline the protocol for defrosting, growing, and preparing tumoral cells for mice inoculation. All steps should be performed in an aseptic environment within a culture hood to avoid external contamination. Clean all surfaces with 70% ethanol before and during work. All media, reagents, and common supplies should be sterile.

Before the experiment, prepare the required medium and solutions to be used in subsequent steps according to the desired experimental setup one day in advance (See catalog number in the key resources table).

1. Thaw tumoral cells.
  a. Select the stocks of triple-negative mouse breast cancer 4T1 cells from the liquid nitrogen tank. Each frozen vial contains 3 × 10^6^ frozen tumoral cells.
  b. Thaw the cells for 1–2 min in a 37°C bath before adding the medium.
    **CRITICAL:** Ensure that all cells are thawed, as uneven thawing can affect cell viability.
  c. Add 1 mL of pre-warmed DMEM/F12 medium supplemented with 6.5% fetal bovine serum.
  d. Transfer 1 mL of cells into sterile tube containing 10 mL of DMEM/F-12 medium.
  e. Centrifuge the tube at 4°C, 225 g for 5 min.
2. Culture tumoral cells.
  a. After centrifugation is complete, carefully aspirate the supernatant using a pipette.
  b. Resuspend cell pellet in 0.5 mL phosphate-buffered saline (PBS) 1X.
  c. Add 3 × 10^6^ cells to a T25 culture flask with 10 mL DMEM/F12 medium and 6.5% fetal bovine serum. This protocol can be scaled up by area and volume, depending on the total tumoral cells needed.
  d. Place culture flask in the incubator at 37°C and 5% CO_2_ atmosphere. Gently loosen the flask cap approximately a quarter turn (semi-open lid) to facilitate gas exchange. Alternatively, use culture flasks with vented caps.
  e. Check cellular morphological features, cell proliferation, membrane integrity, and growth on a daily basis under the microscope.
3. Prepare tumoral cells for mice inoculation.
  a. Remove medium and wash cells twice with sterile 1X PBS.
  b. Add enough Trypsin to completely cover the cells and incubate them at 37°C for 2 min.
  c. Check under the microscope if the cells have detached from the plate.
    **CRITICAL:** It is important to ensure that all cells have detached before proceeding to the next step.
  d. Add 4 mL of DMEM/F12 medium supplemented with 6.5% fetal bovine serum to stop the trypsin reaction. Collect the cells and transfer to a sterile tube.
  e. Centrifuge the cell suspension for 5 min at 4°C, 225 x *g.*
  f. Remove the trypsin solution by aspiration.
  g. Mix the cells with fresh medium containing 6.5% fetal bovine serum.
  h. Determine cell concentration and viability with trypan blue exclusion method using a hemacytometer.^2^
  i. For inoculation, dilute 1 ×10^5^ 4T1 viable cells in 1 mL of PBS 1X.

### Software requirements

**Timing: 15–30 min (for step 7)**

***Note:*** Before starting, ensure you have a conda environment set up with all the necessary dependencies. Furthermore, make sure you have sufficient computational power with multi-threading capabilities to process large datasets, especially during steps like DADA2 denoising and phylogenetic tree construction. Additionally, verify you have enough disk space to store large sequence files and generated artifacts.

4. Install QIIME 2 (v. 2021.4)^3^ in your computer.
  ***Note:*** The native Conda installation is generally the recommended method. Follow the instructions for downloading and installing Miniconda. Here, we describe the installation process for Linux users. For other installation methods and operating systems, please refer to the QIIME 2 documentation.
  a. Open your terminal window and run the following commands.
    #Make sure you are using the latest conda version
    > conda update conda
    #Install wget to download the QIIME 2 installation files
    > conda install wget
  b. Install QIIME 2 and create a conda environment.
    > wgethttps://data.qiime2.org/distro/core/qiime2-2021.4-py38-linux-conda.yml
    #Create the conda environment with the name and version of QIIME 2
    > conda env create -n qiime2-2021.4 --file qiime2-2021.4-py38-linux-conda.yml
  c. Activate conda environment.
    > conda activate qiime2-2021.4
    ***Note:*** In this protocol, we will use QIIME 2 version 2021.4. For information on using other QIIME 2 releases, please refer to the official QIIME 2 website.
5. Download the database for taxa identification using Quantitative Into Microbial Ecology (QIIME) 2 pipeline (v.2021.4).^3^ Go to the page https://docs.qiime2.org/2022.8/data-resources and click on Silva 138 99% OTUs from 515F/806R region of sequences. This will initiate the download of the Silva database.
6. Create a metadata table with all the information about your samples and the conditions of your study (See the Data S1 provided in the Supplemental materials of this protocol). For more information about metadata read here.
7. Install all the necessary packages for R.

> BiocManager::install("decontam")

> BiocManager::install("phyloseq")

> install.packages("ggplot2")

> install.packages("devtools")

> devtools::install_github("vmikk/metagMisc") # Optional, depending on your analysis

> install.packages("openxlsx")

> install_github("zdk123/SpiecEasi")

> install.packages("igraph")

> install.packages("NetSwan")

> install.packages("ALDEx2")

> install.packages("tidyverse")

> install.packages("matrixStats")

> install.packages("Heatplus")

> install.packages("gplots")

> install.packages("vegan")

> install.packages("RColorBrewer")

> install.packages("htmltools")

> install.packages("DESeq2")

## Key resources table

**Table:** 

REAGENT or RESOURCE	SOURCE	IDENTIFIER
**Chemicals, peptides, and recombinant proteins**		

DMEM/F-12 medium	Gibco-BRL	11320033
Fetal bovine serum (FBS)	Gibco-BRL	16000044
Trypsin	Sigma-Aldrich	9002-07-7
Trypan blue solution, 0.4%	Gibco-BRL	15250061
Diazepam	AICA (Havana, Cuba)	1482
Atropine	AICA (Havana, Cuba)	M-17-032-A03
Ketamine	AICA (Havana, Cuba)	M-14-179-N01

**Critical commercial assays**		

NucleoSpin tissue DNA extraction kit	Macherey-Nagel	740952.50
NEBNext Ultra II DNA library prep kit	New England Biolabs	E7645S

**Deposited data**		

16S rRNA gene sequences from mouse feces samples	Wu-Chuang et al.^1^	Bioproject No. PRJNA1008984

**Experimental models: Cell lines**		

4T1 cells	ATCC	CRL-2539

**Experimental models: Organisms/strains**		

6–8 weeks BALB/c mice specific pathogens-free (SPF) mice	CENPALAB (Havana, Cuba)	BALB/c/Cenp

**Software and algorithms**		

Quantitative Into Microbial Ecology (QIIME) 2 pipeline (v.2021.4)	Bolyen et al.^3^	www.qiime2.org
Gephi 0.9.2	Bastian et al.^4^	www.gephi.org
DADA2	Callahan et al.^5^	https://doi.org/10.1038/nmeth.3869
q2-feature-classifier	Bokulich et al.^6^	https://github.com/qiime2/q2-feature-classifier
RStudio	RStudio Team^7^	www.rstudio.com
Decontam package	Davis et al.^8^	https://doi.org/10.1186/s40168-018-0605-2
ggplot2 package	Wickham et al.^9^	https://ggplot2.tidyverse.org
Devtools package	Wickham et al.^10^	https://github.com/r-lib/devtools
metagMisc package	Vladimir Mikryukov^11^	https://github.com/vmikk/metagMisc
openxlsx	Schauberger et al.^12^	https://ycphs.github.io/openxlsx/index.html
SpiecEasi package	Kurtz et al.^13^	https://doi.org/10.1371/journal.pcbi.1004226
Igraph package	Csardi et al.^14^	https://igraph.org
Netswan package	Lhomme S.^15^	https://journals.openedition.org/cybergeo/26763
PICRUSt2 software	Douglas et al.^16^	https://doi.org/10.1038/s41587-020-0548-6
ALDEx2 package	Fernandes et al.^17^	https://doi.org/10.1186/2049-2618-2-15
Tidyverse package	Wickham et al.^18^	https://doi.org/10.21105/joss.01686
matrixStats package	Bengtsson H.^19^	https://github.com/HenrikBengtsson/matrixStats
Heatplus package	Ploner A.^20^	https://github.com/alexploner/Heatplus
gplots package	Warnes et al.^21^	https://github.com/talgalili/gplots
Vegan package	Oksanen et al.^22^	https://vegandevs.github.io/vegan
RColorBrewer package	Neuwirth E.^23^	https://cran.r-project.org/web/packages/RColorBrewer/RColorBrewer.pdf
htmltools package	Cheng et al.^24^	https://github.com/rstudio/htmltools
DESeq2 package	Love et al.^25^	https://doi.org/10.1186/s13059-014-0550-8
Phyloseq package	McMurdie et al.^26^	https://doi.org/10.1371/journal.pone.0061217

**Other**		

Syringe 1 mL with 23G 1′ needle	Terumo	MDSS01SE
Caliper	Mitutoyo	531–103
NanoDrop One	Thermo Scientific	ND2000USCAN
PCR primer 541F: GTGCCAGCMGCCGCGGTAA	Caporaso et al.^27^	https://doi.org/10.1038/ismej.2012.8
PCR primer 806R: GGACTACHVGGGTWTCTAAT	Caporaso et al.^27^	https://doi.org/10.1038/ismej.2012.8

## Step-by-step method details

### Part 1: Establishment of the mouse model of breast cancer

#### Inoculate tumoral cells in mice

**Timing: 10 min per mouse**

This step outlines the protocol for subcutaneously inoculating 4T1 tumoral cells in BALB/c mice.

1. House mice in controlled temperature (20°C–23°C), humidity (65 ± 10%), and a 12-h light-dark cycle that is regulated automatically.
2. Separate randomly two groups of mice: one group will be inoculated with 4T1 tumor cells and the control group without inoculation. Mice from the same group are kept in the same cage (n = 8).
3. Collect the 4T1 tumor cells prepared previously and keep them chilled on ice until the time of injection.

**CRITICAL:** Cells can be kept chilled during 2 hours, after this time viability could reduce. The vial with cells should be shake before inoculation.

4. Weigh the animals of both experimental groups prior to procedure using a precision balance.
5. Tumor inoculation to the inoculated group:
  a. Load one syringe 1 mL with 23G 1′ needle per mouse with 100 μL of 4T1 cells (1 × 10^4^ cells/mouse) in PBS 1x.
    **CRITICAL:** Syringes should be prepared with no bubble.
  b. Hold the mouse gently but firmly by scruff and tail to avoid movement, and clean the ventral area of mouse with 70% ethanol.
  c. Insert the needle perpendicularly into the fourth mammary fat pad (orthotopic implantation).
  d. After needle insertion but prior to the injection (expelling the contends of the syringe), perform the opposite movement and apply light suction force to ensure that needle was not inserted into a vessel.
  e. Gently remove the needle, use a gauze and gentle pressure. Return animal to home cage.
  f. Repeat the steps 5.a to 5.e until all mice have been inoculated.

#### Tumor measurement

**Timing: 18–23 days, depending on tumor latency**

This step outlines the protocol for tumor measurement, starting five days after tumor inoculation.

***Note:*** Clinical signs, symptoms, and the morbidity and mortality of each animal should be assessed daily. Body weight must be measured and recorded weekly using a precision balance following tumor inoculation.

6. Hold the mouse gently but firmly by scruff and tail to avoid movement and gently palpate the tumor inoculation site every alternate day.
7. If tumor is palpable, measure tumor size twice a week using a caliper:
  a. Place the caliper on the axis of the largest diameter of the tumor and adjust the device to the limits of the neoplasia, check the measurement (mm) and repeat the operation for the smaller diameter.
8. Calculate the volume of each individual tumor using the formula: V = πab^2^/6, where 'a' represents the largest tumor diameter and 'b' represents the smaller diameter.
9. Return animal to home cage.
10. Repeat the steps 6 to 8 until finish to examine all mice.

**CRITICAL:** If tumor reaches 2000 mm^3^, tumor breaks through the skin's surface and creates a wound (ulcerated tumor) or mouse evidence pain or suffering, the animal should be euthanized, using cervical dislocation to ensure animal welfare.^28^

#### Mouse feces collection and DNA extraction

**Timing: Variable**

This step explains how to collect mouse feces for microbiota studies. These samples can also be used in additional experimental procedures, such as analysis of endoparasites like helminths and protozoa.

**CRITICAL:** All used materials need to be free of biological material, to avoid DNA contamination.

11. Collect fecal samples from cancer-bearing and healthy control mice at the beginning of the tumor measurement and again when the animal manifest symptoms of disease and begin to die (16- and 22-days post-inoculation (dpi)):
  a. Hold the mouse gently but firmly to prevent movement, positioning its head upwards.
  b. Place the vial near mouse, avoiding contact with animal or your hands. Collect two or three fecal pellets from all animals in both the tumor-inoculated and control groups.
  c. Place the collected samples on ice, then store them at −20°C for immediate use or at −80°C for long-term storage.

***Note:*** Additional samples may be collected at different time points, depending on the objectives of the study.

12. For the DNA extraction, here we describe the procedure using the Nucleospin tissue DNA extraction Kit (See catalog number in the key resources table).
   Lysis of Fecal Samples:
  a. Weigh approximately 25 mg of feces (two or three fecal pellets) of each animal.
  b. Add 180 μL of Buffer T1 (lysis buffer) and 25 μL of Proteinase K solution to the tube.
  c. Mix briefly by vortexing and incubate the samples at 56°C in a still heating block for 12–16 h until the sample is completely lysed.
13. DNA Binding:
  a. Add 200 μL of Buffer B3 to the lysed sample.
  b. Mix thoroughly by vortexing for 15 s.
  c. Incubate the mixture at 70°C for 10 min to complete the lysis process.
  d. Add 210 μL ethanol (96–100%) to the sample and vortex vigorously.
  e. Carefully transfer 700 μL of the supernatant to a NucleoSpin Tissue Column (placed in a collection tube) and centrifuge at 11,000 x g for 1 min.
  f. Discard the flow-through.
14. Washing and Elution:
  a. Add 500 μL of Buffer BW and centrifuge at 11,000 x g for 1 min.
  b. Discard the flow-through.
  c. Load 600 μL of Buffer B5 and centrifuge at 11,000 x g for 1 min.
  d. Discard the flow-through and place the column into a clean 1.5 mL microcentrifuge tube.
15. Elution of DNA:
  a. Add 100 μL of sterile water directly onto the silica membrane.
  b. Incubate at 20°C–23°C for 2 min.
  c. Centrifuge at 13,000 x g for 1 min to elute the DNA and collect the elution.
    **Pause Point:** Samples can be stored at −20°C.
  d. Assess the gDNA concentration and quality (OD_260/280_ between 1.8 and 2.0) using the NanoDrop One spectrophotometer.
    **CRITICAL:** Include at least four extraction reagent controls in which different DNA extraction steps are performed using the same conditions as for the samples, but with water as the template.
    ***Note:*** Each DNA sample was eluted in 100 μL of sterile water.
    ***Note:*** It is possible to use alternative commercial kits for microbiota studies. Examples of suitable kits include the QIAamp DNA Stool Mini Kit (QIAGEN, Dublin, Ireland)^29^ and the Bacteremia DNA Isolation Kit (BiOstic, 12240-50).^30^

#### Evaluate tumor-associated effects

**Timing: 10 min per mouse**

A critical aspect of preclinical study with tumors is the evaluation of tumor-associated effects. This step outlines the endpoint protocol for tumoral studies with animals.

16. Anesthetic treatment:
  a. Weigh the animal prior to procedure, to calculate correct amount of analgesic to administer. Prepare the anesthetic combination of Ketamine/Diazepam/Atropine (50/5/1 mg/kg).
  b. Hold the mouse gently but firmly by scruff and tail to avoid movement, and clean the ventral area of mouse with ethanol 70%.
  c. Put the mouse in a supine position and insert the needle perpendicularly to right pelvic region.
  d. After needle insertion but before injection (expelling the contends of the syringe), do the opposite movement and apply light suction force to ensure that needle insertion was not on the vessels.

**CRITICAL:** Confirm the mouse is deeply anesthetized by checking the lack of pedal reflex, by firmly pinching a foot. No reflexes should be observed.

17. Euthanize the mouse by cervical dislocation.
18. Using surgical scissors, make a small incision in middle line; carefully cutting through the skin to tumor.
19. Dissect carefully the tumor, checking tumors growth in the subcutis and signs of active expansion into nearby environments, such as muscle and dermis. Examine macroscopically abdominal and thoracic organs of mice to detect other effects of the tumor.

### Part 2: Microbiome analysis

#### 16S rRNA gene sequencing

**Timing: Variable**

Here we provide the steps for Illumina sequencing performed at Novogene Bioinformatics Technology Co. (London, UK) (www.novogene.com). Additionally, we describe quality control of sequences, removal of contaminants and taxonomic profiling of microorganisms.

20. Sample preparation:
  a. Prepare the DNA samples in 1.5 mL safe-lock tubes.
  b. Assess the DNA quality using Nanodrop (OD_260/280_ = 1.8–2.0 considered as no degradation or contamination).

***Note:*** At the sequencing facility, samples are required to have at least 200 ng of gDNA with a concentration higher than 10 ng/μL.

**CRITICAL:** The reagent control samples must be included in the sequencing process to remove contaminants from this step later.

21. Library Preparation:
  a. The library preparation for next-generation sequencing can be conducted using the NEBNext Ultra II DNA Library Prep Kit, following the manufacturer’s instructions.

***Note:*** The process includes DNA fragmentation by sonication, end repair, adaptor ligation, and PCR amplification. Once complete, the prepared DNA library's fragment size distribution can be verified using an Agilent 2100 Bioanalyzer.

22. 16S rRNA Gene V4 Region PCR Amplification:
  a. Perform the PCR amplification targeting a specific hypervariable region, such as the V4 region of the 16S rRNA gene, using the bar-coded 515F and 806R universal primers pair.
  b. Execute the reaction in a 25 μL reaction volume containing 12.5 μL of Q5 High-Fidelity DNA Polymerase. Program the reaction with the following thermal cycling parameters: initial denaturation at 95°C for 2 min, 25 cycles of denaturation at 95°C for 30 s, annealing at 55°C for 30 s, and extension at 72°C for 30 s, with a final extension at 72°C for 5 min.

***Note:*** Barcoded primers facilitate the multiplexing of multiple samples in a single sequencing run.

23. Sequencing:
  a. Purify the amplicons for library preparation using the AMPure XP system. Subsequently, perform the quality control with the Qubit 3.0 Fluorometer to ensure accurate pooling for sequencing.
  b. Perform library sequencing in a single lane of Illumina MiSeq with the MiSeq Reagent Kit v2, generating 251-base paired-end reads.

***Note:*** This setup is highly effective in covering the V4 region, thereby enhancing the accuracy of taxonomic profiling.

#### Data processing

**Timing: 1 day**

Here we describe the steps for processing the sequences: quality control, trimming, removal of contaminants, and the generation of the Amplicon Sequence Variant (ASV) table using Quantitative Insights Into Microbial Ecology (QIIME) 2 pipeline (v. 2021.4).^3^

***Note:*** To facilitate the development of the protocol, we will use the data generated by Wu-Chuang et al.^1^ and deposited at the SRA repository (Bioproject No. PRJNA1008984). The data can be downloaded from the SRA Explorer website (www.sra-explorer.info) using the Bioproject number, which allows access to all the FASTQ files for download.

24. Activate the QIIME2 environment and set up the working directory:

> conda activate qiime2-<version>

> cd /your/working/directory/

> export TMPDIR='/your/temp/directory'

***Note:*** Replace `<version>` with your specific QIIME2 version (e.g., `2021.4′) and adjust the directories according to your system.

25. Import the sequence data into QIIME 2. Then generate the summary statistics to verify the sequences and assess the sequencing depth of each sample to ensure data quality:

> qiime tools import \

 --type 'SampleData[PairedEndSequencesWithQuality]' \

 --input-format PairedEndFastqManifestPhred33 \

 --input-path input_manifest.csv \

 --output-path demux.qza

> qiime demux summarize \

 --i-data demux.qza \

 --o-visualization demux.qzv

***Note:*** The `input_manifest.csv` should contain paths to your paired-end sequence files. Further details regarding these files and the creation process can be found here.

26. Denoise sequences using DADA2,^5^ which includes quality filtering, dereplication, and chimera removal. This step requires the metadata table Data S1 from step 6 in the before you begin section:

> qiime dada2 denoise-paired \

 --i-demultiplexed-seqs demux.qza \

 --p-trim-left-f 0 \

 --p-trim-left-r 0 \

 --p-trunc-len-f 200 \

 --p-trunc-len-r 200 \

 --p-n-threads 20 \

 --output-dir dada2

> qiime feature-table summarize \

 --i-table dada2/table.qza \

 --o-visualization table.qzv \

 --m-sample-metadata-file Data_S1.tsv

> qiime feature-table tabulate-seqs \

 --i-data dada2/representative_sequences.qza \

 --o-visualization representative_sequences.qzv

> qiime Metadata tabulate \

 --m-input-file dada2/denoising_stats.qza \

 --o-visualization denoising_stats.qzv

***Note:*** Parameters such as `--p-trim-left` and `--p-trunc-len` can be adjusted based on the quality profiles of your sequences.

27. Taxonomic Classification. Classify the sequences using a pre-trained classifier^6^:

> qiime feature-classifier classify-sklearn \

 --i-classifier silva-<version>-nb-classifier.qza \

 --i-reads dada2/representative_sequences.qza \

 --p-n-jobs 15 \

 --o-classification taxonomy.qza

> qiime Metadata tabulate \

 --m-input-file taxonomy.qza \

 --o-visualization taxonomy.qzv

***Note:*** Replace `<version>` with the appropriate version of the classifier, such as SILVA or Greengenes, depending on your study.

28. Export the feature table and convert it for use in R or other tools:

> qiime tools export \

 --input-path dada2/table.qza \

 --output-path Exported-table

> biom convert -i Exported-table/feature-table.biom -o Exported-table/Table.tsv --to-tsv

29. If contamination is a concern, the `decontam` package^8^ in R for identification and removal:
  a. Load required libraries.
    > library(phyloseq); packageVersion("phyloseq")
    > library(ggplot2); packageVersion("ggplot2")
    > library(decontam); packageVersion("decontam")
    > library(metagMisc) # Optional, depending on your analysis
  b. Import the `Table.tsv` generated in step 28.
    > feature <- read.csv('Table.tsv', header = TRUE, sep = "\t", row.names = 1)
    > taxonomy <- as.matrix(read.csv('Taxonomy.tsv', header = TRUE, sep = "\t", row.names = 1))
    > metadata <- read.csv('Data_S1.tsv', header = TRUE, sep = "\t", row.names = 1)
  c. Create a Phyloseq object using the Phyloseq package^26^ in R.
    > OTU <- otu_table(feature, taxa_are_rows = TRUE)
    > TAX <- tax_table(taxonomy)
    > metadata <- sample_data(data.frame(metadata))
    > ps <- phyloseq(OTU, TAX, metadata)
  d. Inspect library sizes (i.e., number of reads per sample)
    > df <- as.data.frame(sample_data(ps)) # Convert sample data into a ggplot-friendly data frame
    > df$LibrarySize <- sample_sums(ps)
    > df <- df[order(df$LibrarySize), ]
    > df$Index <- seq(nrow(df))
    #Plot library size
    > number_of_reads <- ggplot(data = df, aes(x = Index, y = LibrarySize, color = Stage)), geom_point()
    #Save the plot
    > png("number_of_reads.png")
    > print(number_of_reads)
    > dev.off()
  e. Identify contaminants using the prevalence method.
    > sample_data(ps)$is.neg <- sample_data(ps)$Sample_or_Control == "Control Sample"
    > contamdf.prev <- isContaminant(ps, method = "prevalence", neg = "is.neg")
    # Save the results
    write.csv(contamdf.prev, "contamdf.csv", row.names = FALSE)
    # Examine more stringent threshold for contamination
    > contamdf.prev05 <- isContaminant(ps, method = "prevalence", neg = "is.neg", threshold = 0.5)
    # Examine prevalence in positive and negative samples
    > ps.pa <- transform_sample_counts(ps, function(abund) 1 ∗ (abund > 0))
    > ps.pa.neg <- prune_samples(sample_data(ps.pa)$Sample_or_Control == "Control Sample", ps.pa)
    > ps.pa.pos <- prune_samples(sample_data(ps.pa)$Sample_or_Control == "True Sample", ps.pa)
    # Make data.frame of prevalence in positive and negative samples
    > df.pa <- data.frame(pa.pos = taxa_sums(ps.pa.pos), pa.neg = taxa_sums(ps.pa.neg), contaminant = contamdf.prev$contaminant)
    ***Note:*** For the control samples, utilize the extraction reagent controls in which different all procedure were processed in the same conditions as for the samples, but with water as the template (see steps 12–15).
  f. Remove contaminants.
    > ps.noncontamprev <- prune_taxa(!contamdf.prev$contaminant, ps)
    # Export table
    > write.csv(phyloseq_to_df(ps.noncontamprev), "Prevalence.csv", row.names = FALSE)
    ***Note:*** Adjust `is.neg` based on your metadata, specifically how control samples are labeled.
30. Import the Feature Table into QIIME 2:

***Note:*** In the Supplemental Materials, we have provided an example of metadata table Data S2 required for this step.

> biom convert -i Prevalence-featuretable.txt -o table.from_txt_hdf52.biom --table-type="OTU table" --to-hdf5

> qiime tools import \

 --input-path table.from_txt_hdf52.biom \

 --type 'FeatureTable[Frequency]' \

 --input-format BIOMV210Format \

 --output-path feature-table-decontam.qza

> qiime feature-table summarize \

 --i-table feature-table-decontam.qza \

 --o-visualization table_decontam.qzv \

 --m-sample-Metadata-file Data_S2.tsv

> qiime phylogeny align-to-tree-mafft-fasttree \

 --i-sequences dada2/representative_sequences.qza \

 --p-n-threads 15 \

 --output-dir tree2

31. Diversity Analysis. Perform alpha and beta diversity analyses using QIIME2:

***Note:*** In the Supplemental Materials, we have provided the metadata table Data S2 required for this step.

> qiime phylogeny align-to-tree-mafft-fasttree \

 --i-sequences dada2/representative_sequences.qza \

 --p-n-threads 15 \

 --output-dir tree

> qiime diversity alpha-rarefaction \

 --i-table feature-table.qza \

 --i-phylogeny tree/rooted_tree.qza \

 --m-Metadata-file Data_S2.tsv \

 --p-max-depth <max_depth> \

 --o-visualization alpha-rarefaction.qzv

> qiime diversity core-metrics-phylogenetic \

 --i-table feature-table.qza \

 --i-phylogeny tree/rooted_tree.qza \

 --m-metadata-file Data_S2.tsv \

 --p-sampling-depth <sampling_depth> \

 --output-dir diversity-core

***Note:*** The `--p-max-depth` and `--p-sampling-depth` parameters should be set based on the sequencing depth of your data. To select sample depth, QIIME 2 recommends reviewing the information in `table_decontam.qzv` file (step 30) in the *Interactive Sample Detail* tab (https://view.qiime2.org/). Subsequently, you can choose a value that is as high as possible to retain more sequences per sample, while ensuring that the fewest possible samples are excluded.

32. Collapse and filter taxa for further analysis, such as network analysis:

#To select genus

> qiime taxa collapse \

 --i-table feature-table.qza \

 --i-taxonomy taxonomy.qza \

 --p-level 6 \

 --o-collapsed-table table-level6.qza

> qiime feature-table filter-features \

 --i-table table-level6.qza \

 --p-min-frequency 10 \

 --p-min-samples 3 \

 --o-filtered-table table-level6-filtered.qza

***Note:*** Adjust `--p-level` and filtering criteria (--p-min-frequency and --p-min-samples) based on your analysis needs.

***Note:*** Low abundant taxa can be removed by filtering taxa with less than 10 total reads and present in less than 30% of samples.

33. Export the Filtered Feature Table:

> qiime tools export \

 --input-path table-level6-filtered.qza \

 --output-path Exported-table-level6-filtered

# Convert the BIOM table to a TSV format

> biom convert -i Exported-table-level6-filtered/feature-table.biom -o Exported-table-level6-filtered/Table-level6-filtered.tsv --to-tsv.

#### Differential taxonomic composition

**Timing: 3 h**

Here we provide the steps to determine the relative abundance of taxa using the ALDEx2 package^17^ in R. We also include the steps to visualize the results in R using a heatmap graph.

34. Load required packages and import the ASV/taxa table from the previous step.

>library(ALDEx2)

>library(ggplot2)

>library(tidyverse)

>library(matrixStats)

#import ASV/taxa table

> asv<- read.csv("Table-level6-filtered.tsv", header=T, row.names = 1, sep = '\t')

# filter ASV with less than 20 reads and appearing in less than 3 samples

> asv2 <- asv[rowSums(asv) >= 10, ]

> dim(asv2)

> asv.f <- asv2[rowCounts(asv2 > 0) >= 2, ]

> dim(asv.f)

35. Import the metadata table.

***Note:*** sample.id in rows, make sure the order by sample.id is the same. Additionally, we have provided the Data S3 file in the Supplemental Materials section.

> metadata <- read.csv("Data_S3.tsv", header=T,sep = '\t')

> summary(metadata)

36. Calculate CLR values.

***Note:*** It needs a variable with 2 levels to run aldex.clr (Ex: a column in the metadata file named ‘twolevels’ and set the samples by specific conditions, in this case, 1 for time 1 and 2 for time 2). For an example, see Data S3.

> conds <- as.character(metadata$twolevels)

> a.clr <- aldex.clr(asv, conds, mc.samples=128, denom="all", verbose=F)

> clr.values <- aldex.effect(a.clr, include.sample.summary=TRUE)

> write.csv(clr.values,"clr-values.csv")

37. Run the statistical analysis.

***Note:*** It needs a column in the metadata file named ‘Conditions’ and set the samples by specific conditions that will be compared, in this case, Control_T1 for control groups time 1 and Control_T2 for control groups time 2). For an example, see Data S3 provided in the Supplemental Materials section.

> conds <- as.character(metadata$Condition)

> aldexo <- aldex(asv.f, conds, mc.samples=128, test="t", denom="all", verbose=FALSE)

# Filter output by statistical significance

> aldexo.f <- filter(aldexo, we.eBH < 0.05)

# Add clr values

> aldexo.f$asvID <- rownames(aldexo.f) # adding row names as a column

> clr.values$asvID <- rownames(clr.values) # adding row names as a column

# Merge both tables

> aldexo.clr.f <- merge(aldexo.f, clr.values, by=c("asvID"="asvID"))

# Remove unnecessary columns

> aldexo.clr.f <- select(aldexo.clr.f, -rab.win.1, -rab.win.2, -rab.all, -diff.btw, -diff.win, -effect, -overlap)

# Leave only sample id as column header

> colnames(aldexo.clr.f) <- str_remove(colnames(aldexo.clr.f), "rab.sample.")

head(aldexo.clr.f)

#Export table

write.csv(aldexo.clr.f,"aldex-t-filtered.csv")

38. Visualize using heatmap.
  a. Load libraries and import data.
    library(Heatplus)
    library(gplots)
    library(vegan)
    library(RColorBrewer)
    library(tidyverse)
    # Import dataframe ‘aldex-t-filtered’.
    #This dataframe should only contain as row the asv and as column the clr values. All other columns should be deleted.
    > data1 <- read.csv("aldex-t-filtered.csv", header = TRUE, row.names = 1)
    > data1 <- t(data1)
    > colnames(data1) <- str_remove(colnames(data1), "g__")
    > head(data1)
    #Import metadata
    > metadata1 <- read.csv("Data_S3.tsv", header = TRUE, sep='\t')
    > data <- data1[metadata1$sample.id , ]
    > head(data)
  b. Clustering.
    # To cluster the Taxa:
    Matrix.dist.g <- vegdist(t(data), method = "euclidean")
    col.clus <- hclust(Matrix.dist.g, "aver")
  c. Create the heatmap.
    # Determine the levels
    > metadata <- list()
    > metadata$Condition <- metadata1$Condition
    > metadata$Condition <- factor(metadata$Condition)
    > levels(metadata$Condition)
    # Assign a color to each level in order
    > levels(metadata$Condition)[1] <- "#FFB266"
    > levels(metadata$Condition)[2] <- "#FF8000"
    # Check that each factor got a color
    > levels(metadata$Condition)
    > metadata$Condition <- as.character(metadata$Condition)
    #Choose a scalecolor
    > scalecolor <- colorRampPalette(c("deeppink4","whitesmoke","darkslategray4"), space = "rgb")(100)
    #Look at the heatmap
    > windows()
    > heatmap.2(t(as.matrix(data)),
     Rowv = as.dendrogram(col.clus),
     labRow=as.expression(lapply(colnames(data), function(a) bquote(italic(.(a))))),
     Colv = FALSE,
     na.rm = TRUE,
     col = scalecolor,
     dendrogram = "row",
     trace = "none",
     density.info = "none",
     cexRow = 0.5, cexCol = 0.5,
     lhei = c(2, 2.5),
     key.title = NA,
     key.xlab = "Center Log Ratio",
     margins = c(4, 13),
     ColSideColors = metadata$Condition,
     main = "Cancer")
    > dev.off()
    ***Note:*** Save as PDF or another format. Variables in the last section of the code can be modified.

#### Co-occurrence network analysis

**Timing: 1–2 h**

We provide the co-occurrence network construction using the Sparse Correlations for Compositional Data (SparCC) method, implemented in RStudio, along with Gephi software^4^ for calculating topological features. Steps for keystone taxa identification and network robustness analysis are also described.

39. Co-occurrence network construction:
  a. Import in R the obtained table from QIIME 2:
    # Load required packages
    > library ("openxlsx")
    > library ("SpiecEasi")
    > library ("igraph")
    #Import table
    > table <- as.matrix(read.xlsx("Table-level6-filtered.xlsx", sheet="C1", startRow = 1, colNames = TRUE, rowNames = TRUE, detectDates = FALSE, rows = NULL, cols = NULL, check.names = FALSE, namedRegion = NULL, na.strings = "NA", fillMergedCells = FALSE))
    #Removing empty rows
    > temporal <- rowSums(table)
    > pointer <- which(temporal>0)
    #Make a new table without the empty rows
    > table3 <- table[pointer,]
    > table <- t(table3)
  b. Infer correlations and set a cutoff of 0.75.
    #Inferring correlations
    sparcc.table <- sparcc(table, iter=20, inner_iter=10, th=0.3)
    sparcc.graph <- sparcc.table$Cor
    #Correlation cutoff of 0.75
    sparcc.cutoff <- 0.75
    #Obtain positive and negative interactions
    sparcc.graph <- ifelse(abs(sparcc.table$Cor) >= sparcc.cutoff, sparcc.table$Cor, 0)
    colnames(sparcc.graph) <- colnames(table)
    rownames(sparcc.graph) <- colnames(table)
    diag(sparcc.graph) <- 0
  c. Create igraph objects of each experimental group and export them to “graphml” for Gephi software.
    > ig.sparcc <- graph.adjacency(sparcc.graph, mode = "undirected", weighted = TRUE, diag = FALSE, add.colnames = TRUE)
    #Export
    > write_graph(ig.sparcc, "C1-Cutoff75.graphml", format = c("graphml"))
    ***Note:*** The output file can be imported into Gephi software, where network visualization and the calculation of topological features and taxa connectedness can be performed. In the microbial network, each node represents a bacterial taxon, and each edge represents a co-occurrence correlation. Metrics such has modularity (strength of division of a network into modules), network diameter (shortest path between the two most separated nodes), average degree (average number of links per node), weighted degree (sum of the weight of all the edges connected to a node) and clustering coefficient (degree to which nodes in a network tend to form clusters) can be also determined using Gephi.
40. Keystone taxa identification:

Identify keystone taxa based on the following criteria: (i) high eigenvector centrality, which assesses a node’s importance in a co-occurrence network while accounting for the significance of its neighbors, (ii) ubiquitousness, meaning the bacterial taxa are present across all samples in each condition, and (iii) high abundance.

41. Network robustness analysis.
  ***Note:*** An essential characteristic of microbial co-occurrence networks is their robustness to perturbations, such as the addition or removal of nodes (microbial taxa). Here we provide the steps for performing robustness tests by removing and adding nodes.
  a. Use NetSwan package^15^ in R to simulate the removal of nodes from the network and measure the resulting loss of connectivity.
    ***Note:*** The node removal analysis involves using random or directed attacks. Three strategies can be employed for directed attack: betweenness centrality (removes nodes with the highest betweenness centrality values first), degree centrality (removes nodes with the highest degree centrality values first), and cascading (removes first nodes with the highest betweenness centrality values, and betweenness centrality is recalculated).
    i. Import the edge list of each network from Gephi software.
      #Load required packages
      > library(igraph)
      > library(NetSwan)
      > library(data.table)
      #Import table
      > elec<- read.csv("<edge_list>.csv", header=T)
      > elec<-as.matrix(elec)
      > gra<-graph.edgelist(elec, directed=FALSE)
      ***Note:*** Replace `<edge_list>` with your specific file name of edges.
    ii. Calculate connectivity loss.
      > f4<-swan_combinatory(gra,10)
      > f4<-as.data.frame(f4)
      > setnames(f4, old = c('V1','V2','V3','V4','V5'), new = c('fraction_nodes','loss_connec_BNC','loss_connec_DEG','loss_connec_Cascading','loss_connec_Random'))
    iii. Plot the data.
      > plot(f4[,1],f4[,5], type='o', col='yellow',
        + xlab="Fraction of nodes removed", ylab="Connectivity loss")
      > lines(f4[,1],f4[,3], type='o', col='red')
      > lines(f4[,1],f4[,4], type='o', col='orange')
      > lines(f4[,1],f4[,2], type='o', col='blue')
      > legend('bottomright',c("Random", "Betweenness", "Degree", "Cascading"), lty=c(1,1,1,1), pch=c(1,1,1,1),cex=0.5,
        + col=c("yellow","blue","red", "orange"))
  b. For analyzing the robustness of microbial co-occurrence networks to node addition use the igraph package^14^ in R. In this case we will measure two key network metrics: the size of the Largest Connected Component (LCC) and the Average Path Length (APL).
    i. Import the edge list of each network from Gephi software.
      # Load the required packages
      > library(igraph)
      > library(coin) # For the permutation test
      > library(boot) # For bootstrapping
      > library(stats) # For p.adjust()
      # Load the edge list file
      > edge_list <- read.table("<edge_list>.csv", header = TRUE, sep = ",")
      > edge_list <- edge_list[,-1]
      # Convert node names to character strings
      > edge_list$Source <- as.character(edge_list$Source)
      > edge_list$Target <- as.character(edge_list$Target)
      # Create a vector of all the vertex names
      > all.vertices <- unique(c(edge_list$Source, edge_list$Target))
      # Create the graph object with all vertices
      > g <- graph_from_data_frame(edge_list, directed = FALSE, vertices = all.vertices)
      ***Note:*** Replace `<edge_list>` with your specific file name of edges.
    ii. Calculate the network’s robustness the LCC and APL metrics.
      > n.sim <- 10 # Number of simulations
      > n.add <- 100 # Number of nodes to add in each simulation
      > robustness <- numeric(n.add∗n.sim)
      > path.lengths <- numeric(n.add∗n.sim)
      > new.nodes <- paste0("NewNode", 1:n.add)
      > for (i in 1:n.add) {
       for (j in 1:n.sim) {
       # Add the new nodes and connect them to the existing network
       g.new <- add.vertices(g, i, name = new.nodes[1:i])
       new.edges <- cbind(sample(new.nodes[1:i], i, replace = TRUE), sample(all.vertices, i, replace = TRUE))
       g.new <- add_edges(g.new, new.edges)
       # Calculate the size of the largest connected component (LCC)
       ccs <- clusters(g.new)
       max.cc <- max(ccs$csize)
       robustness[(i-1)∗n.sim+j] <- max.cc
       # Calculate the average path length (APL)
       path.lengths[(i-1)∗n.sim+j] <- average.path.length(g.new)
       }
      }
      # Perform one-sample Wilcoxon signed-rank tests and extract p-values
      > robustness.wilcox <- wilcox.test(robustness, mu = 0, exact = FALSE)
      > path.lengths.wilcox <- wilcox.test(path.lengths, mu = 0, exact = FALSE)
      > robustness.pvalue <- robustness.wilcox$p.value
      > path.lengths.pvalue <- path.lengths.wilcox$p.value
      # Calculate BH adjusted p-values
      > pvalues <- c(robustness.pvalue, path.lengths.pvalue)
      > adjusted_pvalues <- p.adjust(pvalues, method = "BH")
      > robustness.adjusted_pvalue <- adjusted_pvalues[1]
      > path.lengths.adjusted_pvalue <- adjusted_pvalues[2]
      # Perform bootstrapping for confidence intervals
      > boot_func <- function(data, indices) {
      return(mean(data[indices]))
      }
      > robustness.boot <- boot(robustness, boot_func, R = 100)
      > path.lengths.boot <- boot(path.lengths, boot_func, R = 100)
    iii. Fit the linear model and extract the coefficients.
      # Create data frames
      > robustness.df <- data.frame(nodes_added = rep(1:n.add, each=n.sim), robustness = robustness)
      > path.lengths.df <- data.frame(nodes_added = rep(1:n.add, each=n.sim), path_lengths = path.lengths)
      # Fit the linear model and extract the coefficients
      > path_lengths.lm <- lm(path_lengths ∼ nodes_added, data = path.lengths.df)
      > path_lengths_R2 <- summary(path_lengths.lm)$r.squared
      > path_lengths_coef <- coef(path_lengths.lm)[2]
      > path_lengths_pvalue <- coef(summary(path_lengths.lm))[2, "Pr(>|t|)"]
      > robustness.lm <- lm(robustness ∼ nodes_added, data = robustness.df)
      > robustness_R2 <- summary(robustness.lm)$r.squared
      > robustness_coef <- coef(robustness.lm)[2]
      > robustness_pvalue <- coef(summary(robustness.lm))[2, "Pr(>|t|)"]
      # Calculate predicted values from the linear regression model for LCC plot
      > robustness.df$predicted_robustness <- predict(robustness.lm, newdata = robustness.df)
      # Calculate predicted values from the linear regression model for Avg. Path Length plot
      > path.lengths.df$predicted_path_lengths <- predict(path_lengths.lm, newdata = path.lengths.df)
    iv. Calculate the mean of LCC and APL per node added.
      # Mean of Robustness and Path length per node added
      > robustness_mean <- robustness.df[,-3]
      > robustness_m <- aggregate(robustness_mean$robustness, list(robustness_mean$nodes_added), mean)
      > path_mean <- path.lengths.df[,-3]
      > path_m <- aggregate(path_mean$path_lengths, list(path_mean$nodes_added), mean)
      #Export tables
      > write.table(robustness_m, "LCC_with_predictions_mean_<control>.csv", sep = ",", col.names = TRUE, row.names = FALSE)
      > write.table(path_m, "path_lengths_with_predictions_mean_<control>.csv", sep = ",", col.names = TRUE, row.names = FALSE)
      ***Note:*** Replace `<control>` with your specific experimental group name.
      ***Note:*** The results can be visualized in GraphPad Prism 8.0.1.
    v. Export the R squared, coefficient and p values to a CSV file.
      # Create a data frame to store model information
      > LCC_model_info <- data.frame(
      Model = "robustness.lm",
      R_squared = summary(robustness.lm)$r.squared,
      Coefficient = coef(robustness.lm)[2],
      P_value = coef(summary(robustness.lm))[2, "Pr(>|t|)"])
      # Save the model information to a CSV file
      > write.csv(LCC_model_info, "LCC_lm_info_<control>.csv", row.names = FALSE)
      # Create a data frame to store model information
      > Path_model_info <- data.frame(
      Model = "path_lengths.lm",
      R_squared = summary(path_lengths.lm)$r.squared,
      Coefficient = coef(path_lengths.lm)[2],
      P_value = coef(summary(path_lengths.lm))[2, "Pr(>|t|)"])
      # Save the model information to a CSV file
      > write.csv(Path_model_info, "path_lengths_lm_info_<control>.csv", row.names = FALSE)
      ***Note:*** Replace `<control>` with your specific experimental group name.

#### Prediction of functional traits in breast cancer-bearing mouse microbiome

**Timing: 4 h**

We employ the PICRUSt2 pipeline^16^ for the prediction of functional gene abundances based on 16S rRNA gene amplicon sequences.

42. Export the QIIME2 files from the data processing step.

> qiime tools export \

 --input-path dada2/filtered_table.qza \

 --output-path Exported-filtered_table

> qiime tools export \

 --input-path dada2/Filtered_repseqs.qza \

 --output-path Exported-Filtered_repseqs

43. Run the PICRUSt2 pipeline.

#Activate PICRUST2 environment

>conda activate picrust2

# Place sequences in a phylogenetic tree

> place_seqs.py -s Exported-Filtered_repseqs/dna-sequences.fasta -o out.tre -p 10 --intermediate intermediate-place_seqs

# Predict genes and functions (16S)

> hsp.py -i 16S -t out.tre -p 10 -n -o marker_predicted_and_nsti.tsv.gz

# Predict metagenomes with EC (Enzyme Commission) or KO (KEGG Orthology) codes

> hsp.py -i EC -t out.tre -o EC_predicted.tsv.gz -p 10

> hsp.py -i KO -t out.tre -o KO_predicted.tsv.gz -p 10

# Metagenome prediction pipeline

> metagenome_pipeline.py -i Exported-filtered_table/feature-table.biom -m marker_predicted_and_nsti.tsv.gz -f EC_predicted.tsv.gz -o EC_metagenome_out --strat_out

# Extract the file 'pred_metagenome_unstrat.tsv.gz'

> gunzip EC_metagenome_out/pred_metagenome_unstrat.tsv.gz

44. To compare each condition, run the following script in R.
  a. Import and filter the data.
    ***Note:*** To perform this step, we have provided the Data S4 file in the Supplemental Materials.
    #Load libraries
    library(htmltools)
    library(DESeq2)
    library(ggplot2)
    #Import table ‘pred_metagenome_unstrat.tsv’ and metadata
    > countData <- read.csv('pred_metagenome_unstrat.tsv', header = TRUE, sep = "\t",row.names = 1)
    > metaData <- read.csv('Data_S4.tsv', header = TRUE, sep = "\t",row.names = 1)
    #Filter asv with less than 20 reads and appearing in less than 3 samples
    countData <- countData[rowSums(countData) >= 10, ]
    countData <- countData[rowCounts(countData > 0) >= 2, ]
  b. Check the data.
    #check if metaData match with countData
    > all(rownames(metaData) %in% colnames(countData))
    #check if the order of metaData is the same as in countData
    > all(rownames(metaData) == colnames(countData))
    #Put in the same order of metaData the countData
    > countData <- countData[, rownames(metaData)]
    #check again if the order of metaData is the same as in countData
    > all(rownames(metaData) == colnames(countData))
    **CRITICAL:** It is absolutely critical that the columns of the count matrix and the rows of the column data (information about samples) are in the same order. DESeq2 will not make guesses as to which column of the count matrix belongs to which row of the column data, these must be provided to DESeq2 already in consistent order.
  c. Construct DESEQDataSet Object.
    > dds <- DESeqDataSetFromMatrix(countData=countData,
    colData=metaData, design=∼dex)
    #Set the group to which the comparison will be done
    > dds$dex<- relevel(dds$dex, ref = "Control")
    #Run DESEQ function
    > dds <- DESeq(dds)
    #Check result table:
    > res <- results(dds)
    > summary(res)
    #Sort summary list with p-value
    > resOrdered <- res[order(res$padj),]
    #Export the results
    > write.csv(as.data.frame(resOrdered), file="results.csv")
  d. Visualize the results in a volcano plot.
    #reset par
    > par(mfrow=c(1,1))
    # Make a basic volcano plot
    > with(res, plot(log2FoldChange, -log10(pvalue), pch=20, main="Volcano plot", cex=1.5))
    # Add colored points: blue if padj(Put Angle bracket here)0.01, red if log2FC(Put "greater than" here)1 and padj(Put Angle bracket here)0.05)
    > with(subset(res, padj<.05 ), points(log2FoldChange, -log10(pvalue), pch=20, col="blue"))
    > with(subset(res, padj<.05 & abs(log2FoldChange)>2), points(log2FoldChange, -log10(pvalue), pch=20, col="red"))
    #Export dataframe
    > resSig <- subset(res, padj<.05 & abs(log2FoldChange)>2)
    > write.csv(as.data.frame(resSig),
         file="results padj and log2foldchange.csv")

## Expected outcomes

Our protocol provides a clear method for establishing a mouse model of breast cancer for microbiome studies. It delineates the principal stages involved in tumor cell inoculation in mice, fecal sample collection, gDNA extraction, 16S rRNA gene sequencing and data processing.

The 4T1 cells spontaneously produce a highly metastatic tumor, and following inoculation in mice, invasion of neighboring tissues such as muscle and dermis may be expected. The genomic DNA extraction from fecal samples is predicted to yield high-purity DNA with a typical amount ranging from 20 to 35 μg, as indicated by the manufacturer of the commercial kit. In the context of microbiota studies, contamination represents a significant challenge and a major concern. In order to mitigate the impact of microbial contamination, the Decontam package was employed.

This pipeline integrates various methodologies to determine whether breast cancer affects the microbial community in mice. While alpha and beta diversity provide insights into the composition and diversity of the microbiome, the co-occurrence networks serve as a valuable tool for assessing the assembly patterns of the bacterial community. Topological features of the microbial networks offer information about the structure and dynamics of the community, facilitating the identification of keystone taxa at each condition. In a microbial co-occurrence network, each bacterial taxon is represented by a node, with edges indicating the co-occurrence correlation among them. Other topological parameters, such as modularity, can be employed to identify distinct communities or clusters, potentially representing diverse ecological niches or functional groups. Additionally, a larger network diameter indicates that some taxa may be separated by numerous intermediaries, whereas a smaller network diameter suggests that most taxa are closely connected, thereby indicating a more compact network. Regarding average degree, a high value suggests a community where numerous taxa interact extensively with one another, while weighted degree parameter provides insights into which microbial taxa may occupy central roles, based on the strength of their interactions with other taxa. The clustering coefficient indicates a tendency for microbial taxa to form tightly interconnected groups, suggesting the presence of cooperative clusters within the network. Variation in these parameters across different conditions offers insights into potential disruption in the microbial community structure.

The analysis of the network robustness provides insights into the response of microbial networks to perturbations, such as the removal or addition of nodes. Node removal allows the simulation of network resilience by modeling the absence of hub taxa, which can significantly impact network connectivity and structural integrity within the microbiome. Node addition, on the other hand, involves assessing two key parameters: the LCC and the APL. The LCC represents the largest group of mutually reachable microbial taxa, potentially serving as the structural backbone of the community. Conversely, the APL quantifies the average number of steps required to travel between any two nodes in the network, indicating the speed at which information can propagate through the network. A higher value of LCC indicates greater resilience to node addition, whereas higher APL value indicates less efficient information transfer across the network, therefore reducing network robustness. Finally, the prediction of metabolic pathways can provide insight into the metabolic alterations within the bacterial community in the presence or absence of the tumor.

## Limitations

This protocol outlines the principal steps for investigating the microbial community in tumor-bearing mice using fecal samples collected at specific time points. However, this parameter may be subject to variation in accordance with the specific experimental requirements and objectives. Furthermore, the 16 rRNA gene amplicon sequencing method has certain limitations with regards to species-level identification, due to the high degree of homology between closely related species. Consequently, identification is typically limited to the genus level in most cases. Despite the use of the Decontam package in R, environmental contamination remains a significant concern that could affect study outcomes. Regarding the prediction metabolic pathways, the method used in this protocol is based on evolutionary modeling of the gene content from reference genomes. As a result, the accuracy of these predictions is constrained by the extent of current genome databases. Furthermore, this method is unable to differentiate between strain-specific functions within the same species.

## Troubleshooting

### Problem 1

During the 4T1 cell culture, there has been a notable change in the turbidity and color of the culture media (related to steps 1–3 from the before you begin section).

In this case, contamination of the cell culture with bacteria, yeast or fungi is likely the cause. This can occur for a number of reasons, but is typically the result of inadequate aseptic procedures or the use of contaminated reagents.

### Potential solution

When working with animal cells, it is crucial to follow good working practices, thus, ensure you are familiar with these practices before starting. Some potential solutions to this issue include manipulating the cells in a laminar flow hood, wearing gloves, and utilizing a lab coat designated exclusively for the culture room. It is recommended that the necks of bottles and work surfaces be disinfected regularly with a 70% alcohol solution. Check the filters of the hood and ensure that other equipment, including water baths and centrifuges, is free from contamination. It is important to ensure that media, buffers, and sera are obtained from reputable suppliers who can provide assurances about the quality and origin of their products.

### Problem 2

During the tumor measurement, there has been a notable intragroup variability with tumor growth (related to steps 6–8).

In this case, differences in the number of inoculated tumoral cells is likely the cause. This can occur typically due to the sedimentation of tumoral cells during inoculation. Another factor that could be influencing is the subcutaneous inoculation of the animals.

### Potential solution

When inoculating animals with tumoral cells, it is crucial to shake the vial before and during inoculation, thus, homogeneous cellular concentration will be injected in all animals, promoting consistent tumor growth. Additionally, it is recommended to have the proper training in orthotopic mammary administration before starting the procedure. This can help to prevent subcutaneous administration of tumoral cells, taking into consideration that orthotopic tumor grow faster than subcutaneous ones. Sufficient mice should be included in each group to allow for outlier analysis, and animals with larger or reduced tumors identified as outliers should be excluded. Additionally, mice with tumors growing outside the mammary area (e.g., subcutaneous or peritoneal) should also be excluded to maintain study reliability.

### Problem 3

The yield of genomic DNA is too low after DNA extraction (related to steps 12–15).

This problem may arise from several causes, such as an insufficient quantity of starting material, inadequate tissue lysis and homogenization, or the utilization of an inappropriate DNA extraction kit.

### Potential solution

When extracting gDNA from stool, it is important to ensure that the quantity of starting material is correct. Before beginning, please consult the recommended quantities of sample specified in the kit protocol to be employed. In some cases, minimal amount could lead to low DNA quantity, while an excess could result in column clogging and a subsequent reduction in DNA yield.

Incomplete lysis can be avoided by following the instructions provided by the kit manufacturer and using the correct amount of starting sample and appropriate reagents. Additionally, it may be beneficial to extend the lysis incubation period or to conduct an additional incubation step at an increased temperature.

Finally, make sure you are using the appropriate commercial kit for gDNA extraction from fecal samples. Some kits may not be suitable for this process, which could result in poor DNA yield and quality. If you have doubts or experience difficulties with the DNA extraction, it is advisable to consult the technical team of the commercial kit or select an alternative kit that may be more suitable for this task.

### Problem 4

It is not possible to import the sequence data into QIIME 2 (related to step 25).

It is important to note that the QIIME 2 pipeline has the capability to import a variety of FASTQ file types. Therefore, it is essential to ensure that the data file you are importing is correctly identified.

### Potential solution

In this case, you may wish to contact your sequencing facility or collaborator to determine which type of FASTQ data you are using. Once you have the necessary information, you can modify the command provided in step 17 by replacing the option '--type' with the appropriate data type. For further information regarding this issue, please refer to the QIIME 2 documentation at the following link: https://docs.qiime2.org/2024.5/tutorials/importing. This section provides a comprehensive overview of the steps involved in importing various types of FASTQ data into QIIME 2. Should the issue persist or if your data is not addressed in the documentation, we advise you to post your query on the QIIME 2 Forum for assistance.

### Problem 5

The data processing pipeline takes too long to complete (related to steps 24–33).

This problem may arise when running a script that exceeds the expected time for completion. This can occur due to different reasons: 1) resource constraints, such as CPU or Memory (RAM) limitations, 2) processing large datasets, or 3) lack of parallelization, that can lead to sequential processing increasing the time required.

### Potential solution

• Resource constraints: ensure you are meeting the computational requirements for each program. If not, consider switching to a computer with more CPU power, memory, or disk space. If available, using a high-performance computing (HPC) cluster can drastically reduce the time needed for most steps.
• Processing large datasets: Verify that you are using the correct files from your dataset. Check for any duplicate files that could be slowing down the analysis.
• Lack of parallelization: Many steps in QIIME 2 and R can be parallelized, which can significantly reduce processing time. Ensure you’re using the maximum number of threads your system allows.

## Resource availability

### Lead contact

Further information and requests for resources and reagents should be directed to the lead contact Alejandro Cabezas-Cruz (alejandro.cabezas@vet-alfort.fr).

### Technical contact

Technical questions on executing this protocol should be directed to and will be answered by the technical contact Dasha Fuentes (dasha.fuentes@cenpalab.cu) and Alejandra Wu-Chuang (alewch29@gmail.com).

### Materials availability

The reagents, kits, and consumables utilized in the protocol are provided in the “key resources table”.

### Data and code availability

The published article includes all datasets and code generated or analyzed during this study. The data from the 16S rRNA sequences is deposited in the at the SRA repository (Bioproject No. PRJNA1008984).

## Acknowledgments

A.L.C.-A. was supported by the project “CLU-2019-05-IRNASA/CSIC Unit of Excellence” granted by the 10.13039/501100014180Junta de Castilla y León and co-financed by the European Union (10.13039/501100008530ERDF). The graphical abstract/figures were created using Biorender.com.

## Author contributions

A.L.C.-A. and A.W.-C. contributed equally to this work. A.L.C.-A. drafted the original manuscript, organized all the information, and contributed to visualization. A.W.-C., L.A.-D., and A.M. developed detailed scripts and bioinformatics pipelines essential for the study. L.M.-H. designed and detailed protocols for DNA extraction and molecular biology procedures. J.A., A.G., and D.F. developed and refined protocols for the animal model and cell biology experiments. A.C.-C. conceived the study, coordinated the research activities, and supervised the overall project execution.

## Declaration of interests

The authors declare no competing interests.

## Supplemental information

Data S1. Metadata table, related to step 5 of before you begin and step 26 of Step-by-step method details section

Data S2. Metadata table, related to steps 30 and 31

Data S3. Metadata table, related to steps 35–38

Data S4. Metadata table, related to step 44
